# Prognostic impact of adenylyl cyclase-associated protein 2 (CAP2) in glioma: A clinicopathological study

**DOI:** 10.1016/j.heliyon.2020.e03236

**Published:** 2020-01-18

**Authors:** Zahraa Saker, Hisham F. Bahmad, Youssef Fares, Zahraa Al Najjar, Mohamad Saad, Hayat Harati, Sanaa Nabha

**Affiliations:** a Neuroscience Research Center, Faculty of Medical Sciences, Lebanese University, Beirut, Lebanon; b Department of Anatomy, Cell Biology, and Physiological Sciences, Faculty of Medicine, American University of Beirut, Beirut, Lebanon; c Faculty of Medicine, Beirut Arab University, Beirut, Lebanon; d Department of Neurosurgery, Faculty of Medical Sciences, Lebanese University, Beirut, Lebanon; e Faculty of Medical Sciences, Lebanese University, Beirut, Lebanon; f Qatar Computing Research Institute, Hamad Bin Khalifa University, Doha, Qatar

**Keywords:** Cancer research, Neurology, Oncology, Pathology, Extracellular matrix, Nervous system, CAP2, Overexpression, Gliomas, Prognostic biomarker

## Abstract

**Background:**

Gliomas are a group of diseases arising from intracranial neoplastic tissues that produce a wide spectrum of clinicopathological features and morphological changes. Key questions that intrigue neuro-oncology researchers include defining novel oncophenotypic signatures relevant to diagnosing such tumors and predicting prognoses among patients. One of the key regulators of the cellular actin dynamics is adenylyl cyclase-associated protein 2 (CAP2), a protein that has been studied before in the milieu of cancer and shown to be associated with tumor progression; yet, its expression levels in the context of gliomas have not been assessed. Hence, we were interested in investigating CAP2 expression in gliomas and evaluating its clinicopathological and prognostic significance.

**Materials and methods:**

CAP2 expression at the protein level was analyzed in 47 human paraffin-embedded gliomas and normal brain tissues by automated immunohistochemical analysis. Statistical analysis was also performed to assess CAP2 expression level in normal and tumor tissues, and to evaluate its clinicopathological and prognostic significance.

**Results:**

Our results revealed high expression of CAP2 protein in tumors of gliomas compared to normal tissues and normal areas adjacent to tumors. High expression of CAP2 was also associated with advanced tumor grades among gliomas. Kaplan-Meier analysis revealed that high CAP2 expression was associated with poor prognosis of patients with glioma (P < 0.05). In Cox regression analysis, CAP2 expression was indicated as an independent prognostic factor for overall survival (hazard ratio (HR) = 1.843, 95% confidence interval (CI), 1.252–2.714; P < 0.005).

**Conclusion:**

CAP2 is overexpressed in glioma and it is proposed as a potential prognostic biomarker for patients with gliomas. CAP2 expression level may serve as a promising target for diagnosis and treatment of glioma.

## 1 Introduction

Gliomas are highly aggressive cancers with exorbitant morbidity and mortality rates. They are a heterogeneous group of “intracranial neoplasms” that result from the uncontrolled proliferation of cells derived from intracranial tissues and account for about 50% of primary intracranial tumors [1]. Most gliomas share a common clinical presentation and have similar diagnostic approaches as well as initial treatment strategies [2]; yet, they differ in their aggressive behavior. Various oncophenotypic signatures that contribute to the pathophysiology of gliomas might present potential diagnostic and prognostic markers, as well as novel therapeutic targets that could increase survival rate and improve patients' lives.

It is known that the transition from normal into a cancerous state requires dramatic changes in cellular morphology and cytoskeletal organization, loss of polarized organization and cell-cell junctions, and gain of invasive and migratory capabilities [3]. Intense reorganization of the cytoskeleton is involved in the developmental processes of cancer cells [4]. The actin cytoskeleton, one of the elements of the cytoskeleton, is considered a crucial molecular player that demonstrates pivotal roles in several cellular processes such as cytokinesis, motility, and intracellular transport [5]. These processes are crucial for the normal development of multicellular organisms, and their dysregulations might contribute to the development of many diseases [4]. This includes cancer via remodeling mechanisms that are perpetrated by tumor cells to gain invasive and migratory properties [3,6].

The dynamics and organizations of actin filaments are regulated by a variety of actin-binding proteins [7]. One central family of such actin regulators is adenylyl cyclase-associated proteins (CAPs) [8]. CAP is one of the most conserved actin-binding proteins found in all eukaryotic cells [9]. It was first identified in *Saccharomyces cerevisiae* under the name Srv2 that was co-purified with adenylyl cyclase [10]. CAP1 and CAP2 are two isoforms of CAP in mammals with high similarities in their amino acid sequences [11,12]. Their N-terminal domains show low similarity compared to the highly conserved C-terminal domains [13,14]. While CAP1 is well studied and shows a broad expression in nearly all cells and organs, the expression of CAP2 appears to be tissue-specific with significant expression in the brain, heart, skeletal muscles, and skin [12]. This suggests that CAP2 might demonstrate unique functions in these tissues.

Whilst molecular mechanisms of CAPs are still hindered and studies tackling the underlying contributions of those proteins in the context of different diseases are scarce, few studies emphasize its implication in tumorigenesis. For instance, CAP1 has been shown to be overexpressed in pancreatic cancer [15], esophageal squamous cell carcinoma [16], lung cancer [17], hepatocellular carcinoma [18], epithelial ovarian cancer [19], breast cancer [20], and glioma [21,22]. On the other hand, there are few studies on the relation between CAP2 and tumors. However, all the available researches have underlined an overexpression of CAP2 in hepatocellular carcinoma, malignant melanoma, breast cancer and gastric cancer [23]. Nonetheless, and to the best of our knowledge, the status of CAP2 expression has not been addressed in gliomas yet.

Therefore, in the present study, the expression of CAP2 in gliomas compared to normal brain tissues was examined. The association between CAP2 expression level and clinicopathological characteristics was determined, and the prognostic significance of CAP2 in gliomas was evaluated by survival analysis.

## 2 Materials and methods

### 2.1 Patients selection

This retrospective cohort study was conducted using a total of 47 archived formalin-fixed paraffin-embedded brain tissue samples from patients who underwent surgical resection at different hospitals in Lebanon between September 2012 and February 2018. The samples and the clinical-pathological data were provided by Institut National de Pathologie (INP). The study with all its experimental protocols was conducted under the Institutional Review Board (IRB) approvals of the Lebanese University and INP. Approval of the Neuroscience Thesis Committee of the Neuroscience Research Center at the Faculty of Medical Sciences, Lebanese University, was obtained prior to commencement of the study. The work described herein has been carried out in accordance with relevant guidelines and regulations and in agreement with the Code of Ethics of the World Medical Association (Declaration of Helsinki) for experiments involving human subjects.

### 2.2 Patient tissue specimens

Out of the 47 collected brain tissues, 5 normal brain specimens were obtained from patients who received epilepsy surgery and verified for the absence of any tumor by certified pathologist. The remaining 42 samples were glioma tissues and also verified for the absence of any tumor by certified pathologist. Out of these 42 gliomas, 22 normal areas adjacent to tumor tissues were found. Special types of gliomas, such as glioblastoma multiforme, astrocytomas, oligodendrogliomas, and oligoastrocytomas were included in the current study. All the tumors were graded based on the WHO classification system [24]. The average patients' age was 53 years ranging from 12 to 85, and the male to female ratio was 25:17.

### 2.3 Immunohistochemistry (IHC)

The paraffin-embedded tissue micro array sections were deparaffinized, and the subsequent sections were then placed in a Ventana automated immunostaining machine (Ventana Medical Systems Inc., Tucson, AZ). The protocol used for immunohistochemistry staining was based on Ventana Discovery XT, a closed system were all steps are performed inside the instrument, from deparaffinization to counterstaining. All the sections were stained in the same run or in runs following one another. In brief, antigen retrieval was achieved using protease I at 37^o^c for 30 min before applying the primary antibodies. The antibodies used were anti-CAP2 rabbit monoclonal antibody (diluted 1:400; ERP6377, Abcam, Cambridge, UK) and Ki67 anti-human mouse monoclonal antibody (ready to use dilution; PA0118, Leica Biosystem, UK). The visualization system was OptiView DAB and counterstaining with Hematoxylin II and Blue Reagent followed immunostaining. Normal brain tissues were used as positive and negative controls and included in each run of staining; the primary antibody was substituted with non-immune serum for the negative controls. These slides were dehydrated and sealed with coverslips.

### 2.4 IHC evaluation

The whole section in each slide was selected for the analysis by two pathologists in a blinded manner without any knowledge of the clinicopathological characteristics of the patients. The calculated score was based on previously published studies [21,22]. The intensity of CAP2 expression was evaluated and scored in comparison with the control. Negative staining was scored 0. The following scores: 1, 2, and 3 represented a weak, moderate and strong expression of CAP2 respectively. The percentage of CAP2-positive stained cells was scored as: 1 (0–25%), 2 (26–50%), 3 (51–75%) and 4 (76–100%). CAP2 staining level was determined by the immunoreactive score (IRS), which was calculated by multiplying the percentage score. The staining pattern was categorized as low CAP2 expression (IRS, 0–3) and high CAP2 expression (IRS, 4–12).

### 2.5 Statistical analyses

All the statistical analyses were conducted using the IBM SPSS statistical software package version 24.0 (SPSS, Inc., Chicago, IL, USA). The association between CAP2 expression and clinicopathological variables was analyzed by the Pearson χ^2^ test and Fisher's exact test. Spearman's rho correlation coefficient was calculated to determine the relation between CAP2 expression and tumor grade. The survival curve was constructed using the Kaplan-Meier method. Cox regression analysis was performed to predict the factors related to overall survival, taking into consideration that overall survival is the time length from the date of diagnosis of glioma till the death date. A P-value < 0.05 was considered statistically significant in all the study.

## 3 Results

### 3.1 CAP2 mRNA expression patterns in human glioma tissues

**Figure 1:**
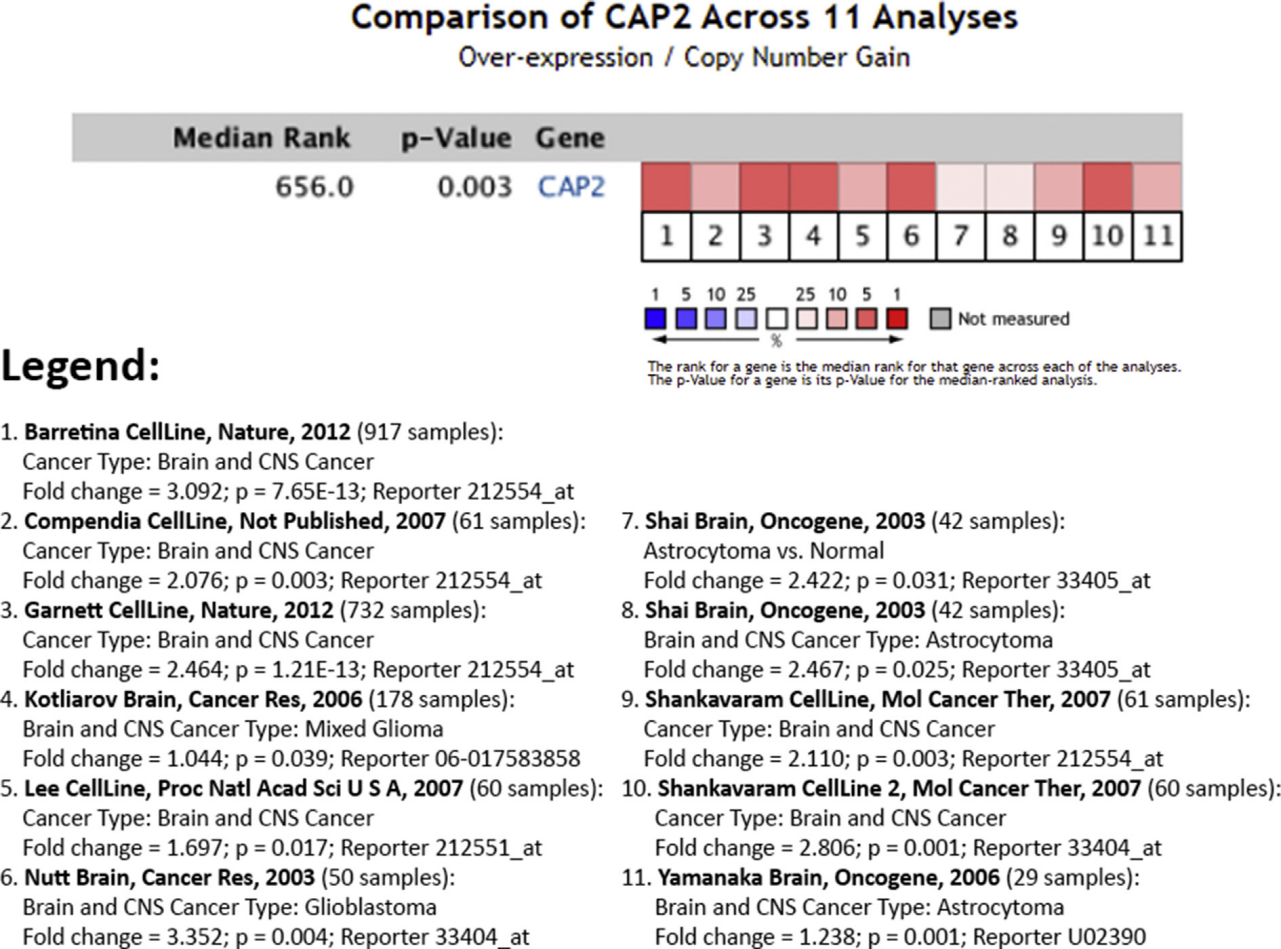
Expression levels of CAP2 mRNA were assessed in 14 sets comprised of human glioma tumor samples. Expression within tumor tissues was presented by fold-change expression of CAP2 and p-values were obtained using t-tests (data retrieved from Ocomine.org). We found statistically significant high expression of CAP2 gene in glioma tissues among the different datasets (fold change ranged between 1.044 and 3.352).

In our study, we first sought to assess the expression patterns of *CAP2* gene in human glioma tumor tissues. We surveyed 11 publicly available datasets (data retrieved from Ocomine.org) comprised of human glioma tumor tissues of different stages and types. Interestingly, analysis revealed that *CAP2* gene was highly expressed in glioma tissues among the different datasets (fold change ranged between 1.044 and 3.352) (Figure 1).

### 3.2 Overexpression of CAP2 in gliomas

**Figure 2:**
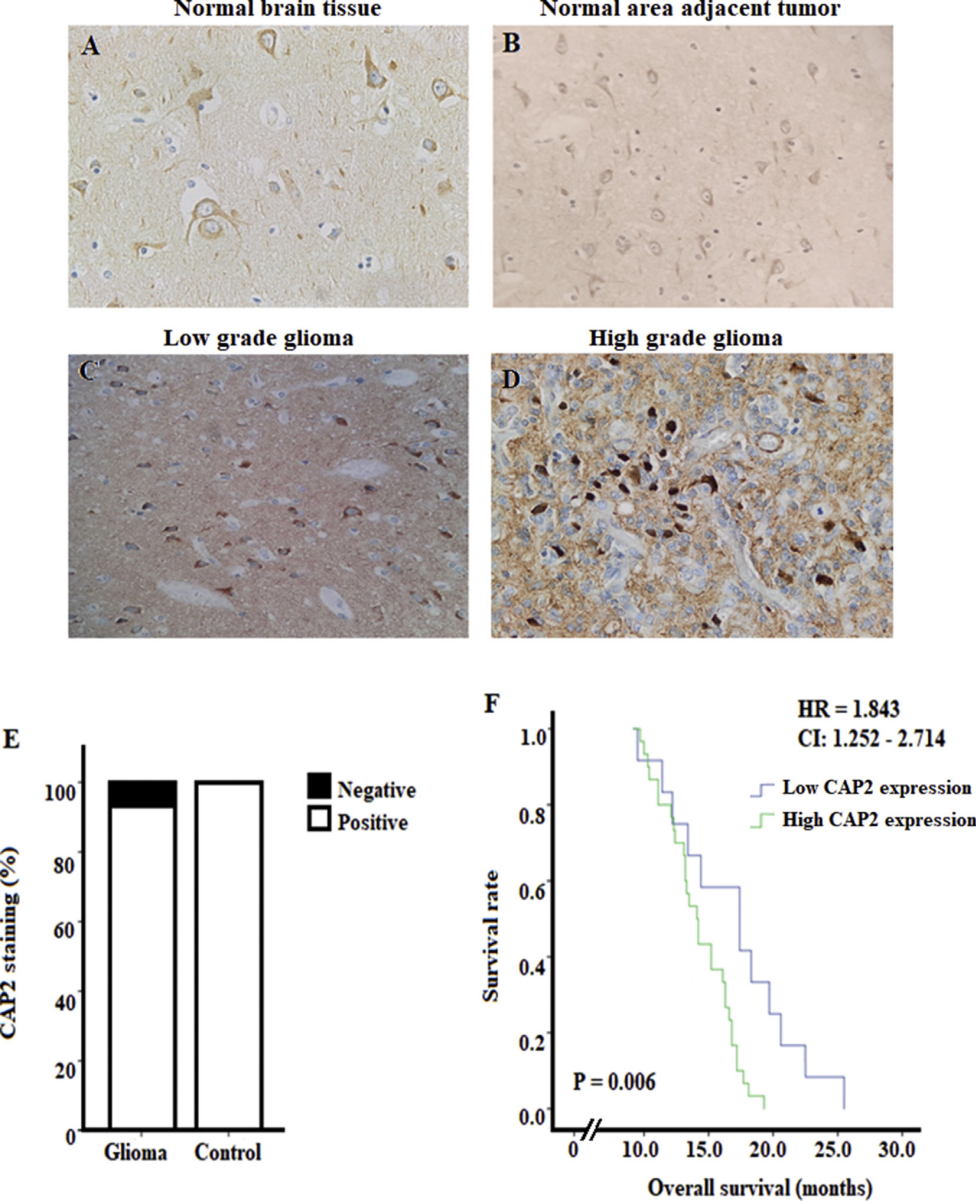
Representative images showing CAP2 immunohistochemical staining in normal and glioma brain tissues, and correlation between survival rate and CAP2 expression in patients with gliomas. (A-D) Positive CAP2 staining in normal brain tissue, normal area adjacent to tumor and in low and high-grade glioma respectively, with more pronounced expression in high-grade gliomas. (E) Positive CAP2 staining in all normal brain tissues and in 92.9% glioma tissues. (F) Survival curve was constructed using the Kaplan-Meier method to study the correlation between survival rate and CAP2 expression in patients with gliomas. Patients were divided into low CAP2 expression (IRS ≤3) and high CAP2 expression (IRS ≥4). Patients with high CAP2 expression had shorter survival than those with low CAP2 expression (P = 0.006). The hazard ratio of CAP2 expression was 1.843 and CI: 1.252–2.714.

For a better understanding of the potential role of CAP2 in gliomas, we investigated and compared the differential expression of CAP2 in glioma and normal brain tissues. As for the normal tissues, CAP2 positive staining was found in all the normal brain tissues and this expression was mainly located at the cell membrane and in the cytoplasm of neurons and glial cells (Figures 2A, 2B, and 2E). However, CAP2 positive staining was observed in 92.9% (39/42) of the glioma tissues (Figure 2E). We also found that CAP2 was mainly located at the cell membrane and in the cytoplasm of tumor cells (Figure 2C).

CAP2 overexpression was observed in 72% (30/42) of glioma tissues. However, low CAP2 protein expression was detected in 96.26% (26/27) normal brain tissues. A significant difference in CAP2 expression was noted between the normal brain tissues and glioma (P < 0.001, χ^2^) with more pronounced expression in tumor specimens.

### 3.3 Association of CAP2 expression and clinicopathological characteristics in gliomas

To investigate the correlation between CAP2 expression and clinicopathological features, the association between CAP2 protein expression and clinicopathological features was analyzed (Table 1). Glioma samples were categorized into two groups according to the expression of CAP2 as having low (IRS ≤3) and high (IRS ≥4) CAP2 expression. No significant differences were observed in CAP2 expression and patients' gender (P = 0.921), age (P = 1.00), resection size (P = 0.695), necrosis (P = 0.195), vessels density (P = 1.00) and Ki67 expression (P = 0.379). However, CAP2 expression was significantly associated with tumor grades (P = 0.014). In addition, Spearman's rho correlation coefficient revealed a positive correlation between tumor grades and CAP2 expression (r = 0.427, P < 0.01).

**Table 1:** CAP2 expression and clinicopathological characteristics of the 42 gliomas.

Characteristics	Total (N = 42) n (%)	CAP2 expression		P-value
		Low (N = 12) n (%)	High (N = 30) n (%)	
Gender				
Male	25 (60)	7 (17)	18 (43)	0.921
Female	17 (40)	5 (12)	12 (28)	
Age				
<55	21 (50)	6 (14)	15 (36)	1.00
≥55	21 (50)	6 (14)	15 (36)	
Tumor Grade				
Low grades (I&II)	7 (17)	5 (12)	2 (5)	0.014*
High grades (III&IV)	35 (83)	7 (17)	28 (66)	
Resection size (cm)				
<3	23 (55)	6 (14)	17 (41)	0.695
≥3	19 (45)	6 (14)	13 (31)	
Necrosis				
Absence	8 (18)	4 (9)	4 (9)	0.195
Presence	34 (45)	8 (18)	26 (26)	
Vessels density				
Normal	3 (7)	1 (2)	2 (5)	1.00
Increased	39 (93)	11 (27)	28 (66)	
Ki67				
Low expression	20 (48)	7 (17)	13 (31)	0.379
High expression	22 (52)	5 (12)	17 (40)	

Statistical analysis was performed by the Pearson χ^2^ test. *P-value < 0.05 was considered significant.

### 3.4 CAP2 expression and prognosis of gliomas

At the end of our study, 31 out of 42 (73.7%) patients died and the remaining 11 (26.2%) patents dropped out of the study. Of the 31 dead patients, 24 (77.4%) patients were in the group of high CAP2 expression and the remaining 7 (22.6%) were in the group of low CAP2 expression. The mean of the follow-up time was 14.98 months ±0.550. Kaplan-Meier analysis showed that patients with high CAP2 expression were accompanied with significantly poorer prognosis in terms of overall survival than those with lower expression (P = 0.006; Figure 2F). Moreover, Cox proportional hazard regression model was constructed including gender, tumor grade CAP2 expression and Ki67 expression. It indicated that the tumor grade (HR, 2.733; CI 1.208–6.181; P = 0.016), CAP2 expression (HR, 1.843; CI, 1.252–2.714; P = 0.002) and Ki67 expression (HR; 1.016; CI, 1.00–1.031; P = 0.044) were independent prognostic variables for patients' overall survival (Table 2). In addition, multiple regression model was used without entering tumor grade variable since we already proved that tumor grade was correlated with CAP2 expression (Part 3.3). This model revealed that high CAP2 expression was correlated with poor prognosis of patients with glioma (r = -0.318, P = 0.041).

**Table 2:** Cox regression analysis in glioma specimens.

	Hazard ratio	95% confidence interval	P-value
Gender	1.139	0.605–2.143	0.687
Tumor grade	2.733	1.208–6.181	0.016*
CAP2 expression	1.843	1.252–2.714	0.002*
Ki67 expression	1.016	1.00–1.013	0.044*

Statistical analysis was performed by the Cox regression analysis. *P-value < 0.05 was considered significant.

## 4 Discussion

Several studies have reported the association between CAP and human cancer. CAP1 has been found to be overexpressed in cancers, including pancreatic, lung, esophageal, liver, ovarian, breast, and glioma cancers [15–21]. However, few studies have focused on the role of CAP2 in human cancer. Shibata *et al.* reported overexpression of CAP2 in hepatocellular carcinoma and found a correlation between levels of expression and multistage hepato-carcinogenesis [25]. Besides, Masugi *et al.* found that CAP2 expression in melanoma appears to increase stepwise during tumor progression. Their results suggest that CAP2 might be a useful prognostic marker in malignant melanoma [26]. It has been also reported that CAP2 is overexpressed in breast cancer, and it was significantly associated with progesterone receptor expression and decreased overall survival, thereby might be considered as a biomarker for the prediction of breast cancer prognosis and survival [27]. Moreover, CAP2 had been found to be upregulated in gastric cancer and was associated with lymph node and distant metastases, and considered as a molecular marker of gastric cancer [23]. However, to date, the prognostic implication of CAP2 in gliomas has not been analyzed.

In our study, CAP2 was obviously found in the cytoplasm and cell membranes among normal neurons and glial cells and tumor cells. Our result is consistent with previous findings that reported the cytoplasmic and membranous localization of CAP2. For example, in breast cancer, CAP2 expression was mainly localized in the cytoplasm of tumor cells, with some CAP2 subsiding in the nucleus [27]. In hepatocellular carcinoma, CAP2 expression was observed in the cytoplasm [28] and the membranous or apical portion of progressed HCC in pseudoglandular patterns [25]. Melanoma cells revealed cytoplasmic CAP2 expression with no detectable staining among normal melanocytes [26]. Besides, CAP2 expression was mainly localized in the cytoplasm of gastric cancer cells and weakly expressed in noncancerous gastric tissues [23].

Our current data showed that CAP2 is significantly overexpressed at protein levels in gliomas compared to normal brain tissues and normal areas adjacent to tumors using IHC assay. Molecular markers are widely used to make precise diagnosis especially when the histologic diagnosis is very difficult. Thus, we suggested that CAP2 might serve as a potential prognostic biomarker for gliomas. This is in consistence with other studies that suggested CAP2 as a marker for malignant melanoma, breast cancer, and gastric cancer [23,26,27]. Furthermore, high CAP2 expression in gliomas was significantly associated with higher grades tumors (grade III and IV) compared to low-grade tumors (grade I and II). This demonstrated that CAP2 might play an important role in the development and prognosis of gliomas.

We further analyzed the relationship between CAP2 expression and the clinicopathological characteristics of patients with gliomas. High CAP2 expression was significantly associated with advanced tumor grade. Meanwhile, there were no significant associations between CAP2 expression and other clinicopathological features. Earlier studies confirmed that CAP1, a homolog of CAP2, is overexpressed in many cancers, and its expression was correlated with tumor grades in human epithelial ovarian cancer and glioma [19,21], and with the degree of malignancy in glioma [22]. The present study demonstrated that high CAP2 expression level was associated with poor overall survival in patients with gliomas. We proposed that CAP2 might be implicated in tumor progression by facilitating intracellular transport, proliferation, cell migration, and invasion, as shown in other tumors [28,29]. Statistical analysis indicated that CAP2 is an independent prognostic factor in gliomas. Interestingly, our results suggested the possibility of using CAP2 as a predictor for survival and prognosis.

## 5 Limitations

We believe that our pilot study has some limitations. First, although this is the first translational research paper to evaluate the expression patterns of CAP2 in human glioma tumor tissues, the sample size is relatively small and hence, the results obtained require conducting subsequent studies on a larger cohort. In accordance, we believe that more data and follow up is required to assess the correlation of CAP2 expression with clinical outcomes and to compare CAP2 expression among the different types of glioma tumors as well. Second, we used in our study a small number of normal brain specimens as a control. In fact, only 5 specimens were acquired since obtaining normal brain tissue is indeed challenging and brain resection is usually done only in limited cases as in epilepsy patients or patients with brain tumors who need surgical resection. So, we collected in our study 5 brain specimens from epilepsy patients, and these tissues were verified for the absence of any tumor by a pathologist, and we also obtained and analyzed the normal areas adjacent to tumor from 22 of our glioma patients whom specimens were verified for the absence of tumor, ending up with 27 normal brain tissues.

## 6 Conclusions

In conclusion, we demonstrated, that CAP2 was noticeably overexpressed in gliomas compared with normal tissues. High CAP2 expression is suggestive of the involvement of this protein in multistep carcinogenesis of gliomas. Although our study is retrospective in nature and the sample size is small, the obtained results propose CAP2 as a promising molecular biomarker for gliomas. Further studied are indeed required to enrich the molecular mechanisms underlying roles of CAP2 in gliomas.

## Declarations

### Author contribution statement

H. F. Bahmad: analyzed and interpreted the data; wrote the paper.

Z. Saker: performed the experiments; analyzed and interpreted the data; wrote the paper.

Y. Fares, S. Nabha, H. Harati: conceived and designed the experiments.

Z. Al Najjar: performed the experiments; analyzed and interpreted the data.

M. Saad: analyzed and interpreted the data.

### Funding statement

This work was supported by funding from the Neuroscience Research Center, Faculty of Medical Sciences, Lebanese University, Beirut, Lebanon. The funders had no role in study design, data collection, and analysis, decision to publish, or preparation of the manuscript.

### Competing interest statement

The authors declare no conflict of interest.

### Additional information

No additional information is available for this paper.
